# How γ-secretase hits a moving target

**DOI:** 10.7554/eLife.20043

**Published:** 2016-08-31

**Authors:** Charles R Sanders

**Affiliations:** Department of Biochemistry, Vanderbilt Univeristy, Nashville, United Stateschuck.sanders@vanderbilt.edu

**Keywords:** gamma-secretase, amyloid-beta peptides, intramembrane protease, Alzheimer's disease, presenilin, processive, C99, Other

## Abstract

An improved understanding of the ways that amyloid-beta peptides are formed could help efforts to find a treatment for Alzheimer’s disease.

**Related research article** Bolduc DM, Montagna DR, Seghers MC, Wolfe MS, Selkoe DJ. 2016. The amyloid-beta forming tripeptide cleavage mechanism of γ-secretase. *eLife* **5**:e17578. doi: 10.7554/eLife.17578

γ-Secretase is a protease enzyme that can cleave a wide range of different transmembrane proteins. One of these is a protein called C99 that is involved in the production of the Aβ polypeptides that are thought to lead to Alzheimer’s disease (Selkoe and Hardy, 2016). C99 contains 99 amino acid residues in three domains: the amyloid intracellular domain extends into the cytosol; the transmembrane domain, which has a helical structure, is embedded in the membrane that surrounds the cell or one of the many subcellular compartments within it; and the N-terminal domain extends outside the cell or into a subcellular compartment (Figure 1).

**Figure 1.**
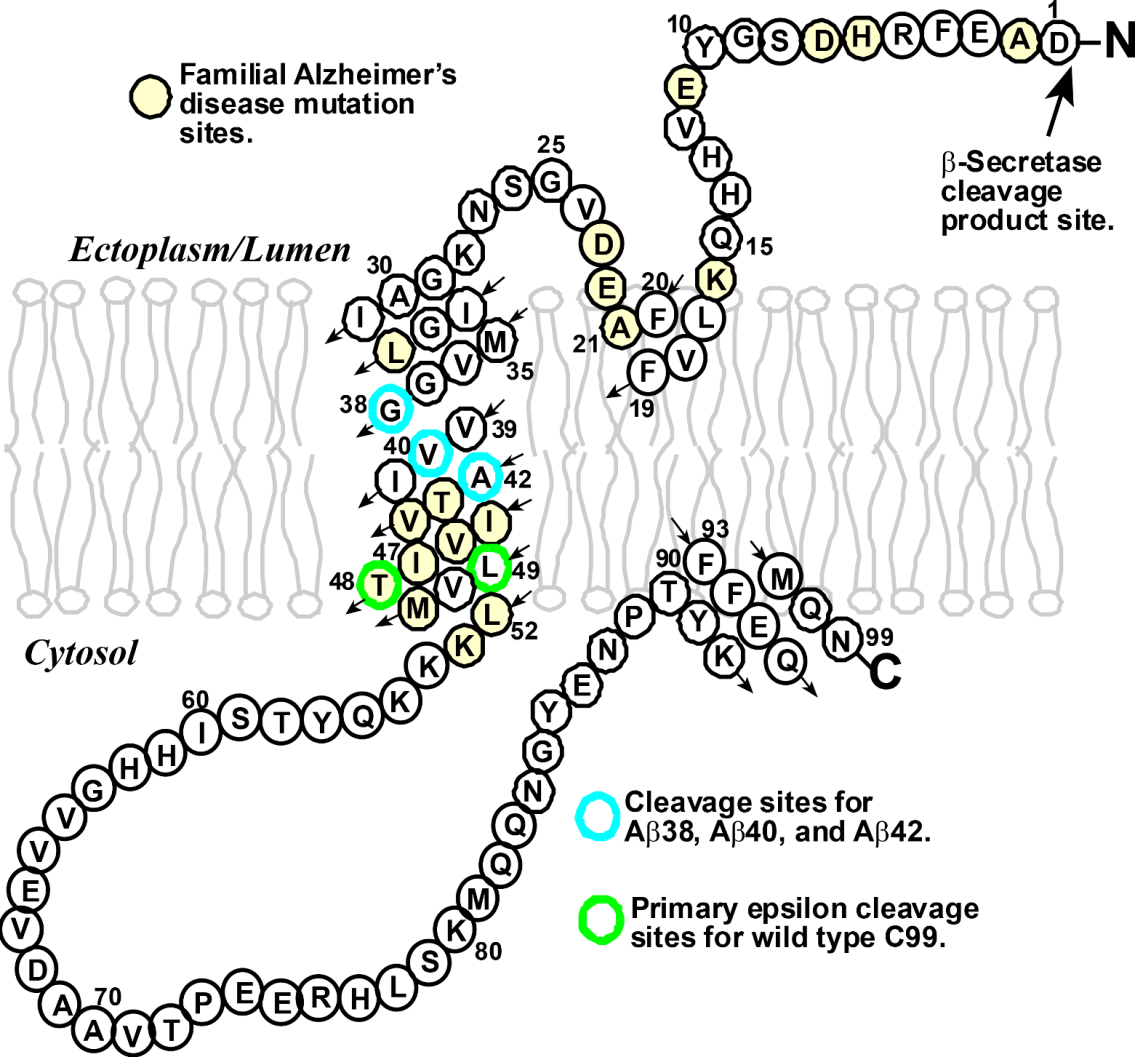
The structure of the human amyloid precursor protein C99. C99 is a transmembrane protein that contains 99 amino acids derived from β-secretase cleavage of the amyloid precursor protein. Mutations at certain amino acid sites (highlighted in yellow) can lead to the inherited form of Alzheimer’s disease. Wild-type C99 is normally first cleaved by the γ-secretase enzyme after site 48 or 49 (green), followed by additional "processive cleavage" events that shorten the polypeptide (which is still bound to the membrane) . The polypeptide is released from the membrane by a final cleavage event after one of the sites highlighted in blue. The small arrows indicate the direction of the amino acid chain in an N- to C-terminal manner.

Years of patient toil by a number of labs have revealed that the γ-secretase enzyme acts by first cleaving C99 near the cytosolic end of its transmembrane domain (Langosch et al., 2015; Morishima-Kawashima, 2014). This “epsilon cleavage” reaction releases the amyloid intracellular domain into the cytosol, leaving behind a amyloid-beta (Aβ) polypeptide that is still bound to the membrane. The γ-secretase enzyme then starts to shorten this Aβ polypeptide – which usually contains either 48 or 49 residues – by "clipping off" short peptides (typically containing just three residues) in a process known as "processive cleavage". Once the length of the Aβ polypeptide has been shortened to between 38 and 43 residues, it is released from the membrane.

Understanding the actions of the γ-secretase enzyme is extremely important because small differences in the lengths of the Aβ polypeptides are thought to have crucial roles in several forms of Alzheimer’s disease. In general, an Aβ polypeptide that starts with 49 residues is shortened by the γ-secretase enzyme to one with 40 (which is called Aβ_40_), and a polypeptide that starts with 48 residues is shortened to one with 42 (Aβ_42_). The production of too much Aβ_42_, relative to Aβ_40_, is associated with the rare inherited forms of Alzheimer’s disease; high levels of Aβ_42_ have also been linked to the more common sporadic form of the disease (Gregory and Halliday, 2005). There is, accordingly, a clear need for drugs that can reduce the production of Aβ polypeptides overall, and also for drugs that can modulate the cleavage of C99 to reduce the production of Aβ_42_ relative to Aβ_40_ (Figure 1). The Aβ_42_ polypeptides cause Alzheimer’s disease by forming highly toxic oligomers that go on to form immunogenic amyloid deposits.

Now, in eLife, Dennis Selkoe, Michael Wolfe and co-workers – including David Bolduc as first author, Daniel Montagna and Matthew Seghers – report results that improve our understanding of the competing reaction pathways that lead to the production of Aβ polypeptides of different length (Bolduc et al., 2016). A central result is that the various cleavage reactions of C99 by the γ-secretase enzyme depend on the properties of the three residues after the cleavage site (which are called the S1', S2' and S3' sites). In particular, the γ-secretase enzyme cannot cleave C99 after a given site if the amino acid at its S2' site is aromatic. Since wild-type C99 does not have any aromatic amino acids in its transmembrane domain, there are no absolute sequence restrictions on where a cleavage event can take place. However, if genetic techniques are used to replace the amino acid at, say, site 50, with an aromatic amino acid, then the γ-secretase enzyme cannot cleave C99 after site 48. Bolduc et al. – who are based at Brigham and Women's Hospital and Harvard Medical School – took advantage of this to make a number of other important observations.

To begin, for wild-type C99 under both purified and cellular conditions, it was confirmed that epsilon cleavage after residue 49 results in the production of Aβ_40_, and that epsilon cleavage after residue 48 results in the production of Aβ_42_. It was also shown that introducing aromatic mutations in residues *before* the normal cleavage sites at residues 48 and 49 can have two effects. First, these mutations can change the relative probability that C99 will be cleaved after site 48 or site 49. (This ratio is normally sensitive to the exact experimental conditions: however, introducing aromatic mutations before these two sites can change this ratio for a given set of experimental conditions). Second, these mutations can also disrupt the normal Aβ_48_–> Aβ_45_–>Aβ_42_ and Aβ_49_–>Aβ_46_–>Aβ_43_–>Aβ_40_ reaction pathways (thus “uncoupling” the connection between the initial epsilon cleavage event and the subsequent processive cleavage events). This is an important observation that provides insight into how certain mutations in C99 that cause the inherited form of Alzheimer’s disease can increase the Aβ_42_-to-Aβ_40_ ratio.

Bolduc et al. also showed that replacing the amino acids at sites 50 and 51 (which are the S2’ positions for the two normal epsilon cleavage sites) with phenylalanine (which is aromatic) blocked the normal epsilon cleavage reactions and, surprisingly, activated epsilon-like cleavage after site 47 instead. The resulting Aβ_47_ was then shortened by γ-secretase to produce Aβ_40_, confirming that processive cleavage sometimes involves the "clipping off" of short peptides that contain four rather than three residues (Takami et al., 2009).

Moreover, Bolduc et al. found that replacing entire tracts of residues in the lower transmembrane domain of C99 with aromatic amino acids resulted in cleavage after site 38, which is near the middle of the domain – a shift of some 10–11 residues from the normal epsilon cleavage sites! This provides significant insight because site 37 and site 38 both contain the amino acid glycine (Gly), and it is known that this double-glycine motif destabilizes the helix in the transmembrane domain (Figure 1; Barrett et al., 2012). This suggests that the initial epsilon cleavage site must be part of a destabilized helix. For wild-type C99 the proximity of the lower end of the transmembrane domain to both the cytosol and to a stop motif formed by three lysine amino acids at sites 53–55 almost certainly leads to a transient fraying of the helix there. If the two normal epsilon cleavage sites (48 and 49) are blocked by an aromatic residue at the S2' position, there is still enough fraying for site 47 to be a viable alternative site. And if the whole lower transmembrane domain is blocked, C99 can still form a complex with γ-secretase, and this allows the enzyme to recognize the destabilization of the helix caused by the double glycine motif, which leads to cleavage after site 38.

For many enzymes, the initial binding event leads directly to the substrate occupying the catalytic site of the enzyme, poised for the chemical reaction. Bolduc et al. found that the γ-secretase enzyme was different: a second step is needed. This result is supported by previous studies of γ-secretase with active site-directed inhibitors (see, for example, Li et al., 2014). Indeed, the γ-secretase enzyme is similar in many ways to another protease enzyme, rhomboid (Cho et al., 2016), even though there appears to be no evolutionary relationship between the two. It seems as if Nature has converged on mechanistic traits that are shared by otherwise unrelated intramembrane proteases. These traits appear to include the following: control of water access to active sites that are buried inside membrane; different mechanisms for initial substrate binding and formation of the catalytic complex; and the ability to scan bound transmembrane segments for suitable cleavage sites (Baker and Urban, 2012; Cho et al., 2016; Dickey et al., 2013; Langosch et al., 2015).

The work of Bolduc et al. represents a major advance in our understanding of catalysis by the γ-secretase enzyme. And while many questions remain unanswered, the availability of near-atomic resolution structures for both C99 (Barrett et al., 2012) and γ-secretase (Bai et al., 2015a; Bai et al., 2015b; Lu et al., 2014) means that further advances are likely to follow as researchers combine biochemical results with structural data and insights.

## References

[bib1] Bai XC, Rajendra E, Yang G, Shi Y, Scheres SH (2015a). Sampling the conformational space of the catalytic subunit of human γ-secretase. eLife.

[bib2] Bai XC, Yan C, Yang G, Lu P, Ma D, Sun L, Zhou R, Scheres SH, Shi Y (2015b). An atomic structure of human γ-secretase. Nature.

[bib3] Baker RP, Urban S (2012). Architectural and thermodynamic principles underlying intramembrane protease function. Nature Chemical Biology.

[bib4] Barrett PJ, Song Y, Van Horn WD, Hustedt EJ, Schafer JM, Hadziselimovic A, Beel AJ, Sanders CR (2012). The amyloid precursor protein has a flexible transmembrane domain and binds cholesterol. Science.

[bib5] Bolduc DM, Montagna DR, Seghers MC, Wolfe MS, Selkoe DJ (2016). The amyloid-beta forming tripeptide cleavage mechanism of γ-secretase. eLife.

[bib6] Cho S, Dickey SW, Urban S (2016). Crystal structures and inhibition kinetics reveal a two-stage catalytic mechanism with drug design implications for rhomboid proteolysis. Molecular Cell.

[bib7] Dickey SW, Baker RP, Cho S, Urban S (2013). Proteolysis inside the membrane is a rate-governed reaction not driven by substrate affinity. Cell.

[bib8] Gregory GC, Halliday GM (2005). What is the dominant Aβ species in human brain tissue? A review. Neurotoxicity Research.

[bib9] Langosch D, Scharnagl C, Steiner H, Lemberg MK (2015). Understanding intramembrane proteolysis: from protein dynamics to reaction kinetics. Trends in Biochemical Sciences.

[bib10] Li Y, Lu SH, Tsai CJ, Bohm C, Qamar S, Dodd RB, Meadows W, Jeon A, McLeod A, Chen F, Arimon M, Berezovska O, Hyman BT, Tomita T, Iwatsubo T, Johnson CM, Farrer LA, Schmitt-Ulms G, Fraser PE, St George-Hyslop PH (2014). Structural interactions between inhibitor and substrate docking sites give insight into mechanisms of human PS1 complexes. Structure.

[bib11] Lu P, Bai XC, Ma D, Xie T, Yan C, Sun L, Yang G, Zhao Y, Zhou R, Shi Y, Shi Y, Yang LF, Zhao GH, Zhou YY (2014). Three-dimensional structure of human γ-secretase. Nature.

[bib12] Morishima-Kawashima M (2014). Molecular mechanism of the intramembrane cleavage of the β-carboxyl terminal fragment of amyloid precursor protein by γ-secretase. Frontiers in Physiology.

[bib13] Selkoe DJ, Hardy J (2016). The amyloid hypothesis of Alzheimer's disease at 25 years. EMBO Molecular Medicine.

[bib14] Takami M, Nagashima Y, Sano Y, Ishihara S, Morishima-Kawashima M, Funamoto S, Ihara Y (2009). γ-Secretase: successive tripeptide and tetrapeptide release from the transmembrane domain of β-carboxyl terminal fragment. Journal of Neuroscience.

